# Prolonged Withdrawal From Escalated Oxycodone Is Associated With Increased Expression of Glutamate Receptors in the Rat Hippocampus

**DOI:** 10.3389/fnins.2020.617973

**Published:** 2021-01-18

**Authors:** Aaron J. Salisbury, Christopher A. Blackwood, Jean Lud Cadet

**Affiliations:** National Institute on Drug Abuse, Molecular Neuropsychiatry Branch, National Institutes of Health, Baltimore, MD, United States

**Keywords:** hippocampus, incubation of drug craving, NMDA receptor, AMPA receptor, opioid use disorder, metabotropic glutamate receptor

## Abstract

People suffering from opioid use disorder (OUD) exhibit cognitive dysfunctions. Here, we investigated potential changes in the expression of glutamate receptors in rat hippocampi at 2 h and 31 days after the last session of oxycodone self-administration (SA). RNA extracted from the hippocampus was used in quantitative polymerase chain reaction analyses. Rats, given long-access (9 h per day) to oxycodone (LgA), took significantly more drug than rats exposed to short-access (3 h per day) (ShA). In addition, LgA rats could be further divided into higher oxycodone taking (LgA-H) or lower oxycodone taking (LgA-L) groups, based on a cut-off of 50 infusions per day. LgA rats, but not ShA, rats exhibited incubation of oxycodone craving. In addition, LgA rats showed increased mRNA expression of *GluA1-3 and GluN2a-c* subunits as well as *Grm3*, *Grm5*, *Grm6*, and *Grm8* subtypes of glutamate receptors after 31 days but not after 2 h of stopping the SA experiment. Changes in *GluA1-3, Grm6, and Grm8* mRNA levels also correlated with increased lever pressing (incubation) after long periods of withdrawal from oxycodone. More studies are needed to elucidate the molecular mechanisms involved in altering the expression of these receptors during withdrawal from oxycodone and/or incubation of drug seeking.

## Introduction

The opioid epidemic that includes the abuse of oxycodone is associated with large numbers of overdose-related deaths (Wilson et al., 2020). Oxycodone is a semisynthetic opioid analgesic prescribed to patients suffering from moderate to severe pain (Riley et al., 2008). Oxycodone use disorder (OUD) is a chronic relapsing disorder characterized by compulsive drug taking despite adverse life consequences (DSM-V, 2013). In people with OUD, neurocircuits in the brain’s reward systems that control hippocampus-mediated cognitive processes including learning and memory (Cadet et al., 2014a) are altered (Koob and Volkow, 2010). Cognitive processes are indeed affected in patients who abuse opioids (Kroll et al., 2018; Allegri et al., 2019).

Although the hippocampus is essential for cognitive functions that can be disturbed in substance use disorders (SUDs), it has received much less attention than other brain regions such as the nucleus accumbens or dorsal striatum in studies involving animal models of SUDs. Nevertheless, the hippocampus has been shown to be important in the regulation of drug intake (Glick and Cox, 1978; Chambers and Taylor, 2004; Brady et al., 2010) and to mediate context- and cue-induced reinstatement of drug taking after withdrawal (Fuchs et al., 2005; Rogers and See, 2007). Importantly, alcohol and opioid exposure negatively impact adult hippocampal neurogenesis (Zhang et al., 2016) and enhances long-term potentiation (LTP; Elahi-Mahani et al., 2018). Furthermore, there is evidence to show that the strength of hippocampal inputs into the nucleus accumbens can bidirectionally drive motivation for rewarding stimuli (LeGates et al., 2018). While these studies have shown a significant role for the hippocampus in mediating drug taking and re-instatement, there is not enough research that documents the effects of opioid drugs on gene expression in the hippocampus. In order to develop more effective opioid addiction treatments, it is necessary to identify molecular neuroadaptations that occur in the hippocampus during long-term exposure and withdrawal from these drugs. To reach these aims, we have used a rat oxycodone self-administration (SA) model to probe the potential molecular changes that occur in that brain region.

The present study was designed to identify potential changes in the mRNA expression of several glutamate receptor subunits in the hippocampus of rats that had been exposed to oxycodone during drug SA experiments. So far, there had been no studies that examined changes in the expression and/or compositions of α-amino-3-hydroxy-5-methyl-4-isoxazolepropionic acid receptors (AMPARs) and N-methyl-D-aspartate receptors (NMDARs), both of which are important for the induction and maintenance of LTP, a process that is impacted by opioids (Terman et al., 1994; Portugal et al., 2014). It is also to be noted that metabotropic glutamate receptors (mGluRs) have also been implicated in animal models of SUDs (Olive, 2009).

Herein, we report that long-term withdrawal from long-access (LgA) to oxycodone is associated with selective increases in AMPAR and NMDAR subunits glutamate receptors in the rat hippocampus. Some subunits of Group I and Group III metabotropic receptors were also affected.

## Experimental Procedures

### Subjects

Male Sprague–Dawley rats, (Charles River Laboratories, Raleigh, NC, United States) weighing 350–400 g, were housed singly prior to surgery on a 12-h light/dark cycle and had food and water freely available. All procedures were performed according to guidelines outlined in the eighth Edition of National Institutes of Health (NIH) Guide for the Care and Use of Laboratory Animals and were approved by the local National Institute of Drug Abuse Intramural Research Program, Animal Care and Use Committee (ACUC).

### Intravenous Surgery and Self-Administration Training

**FIGURE 1:**
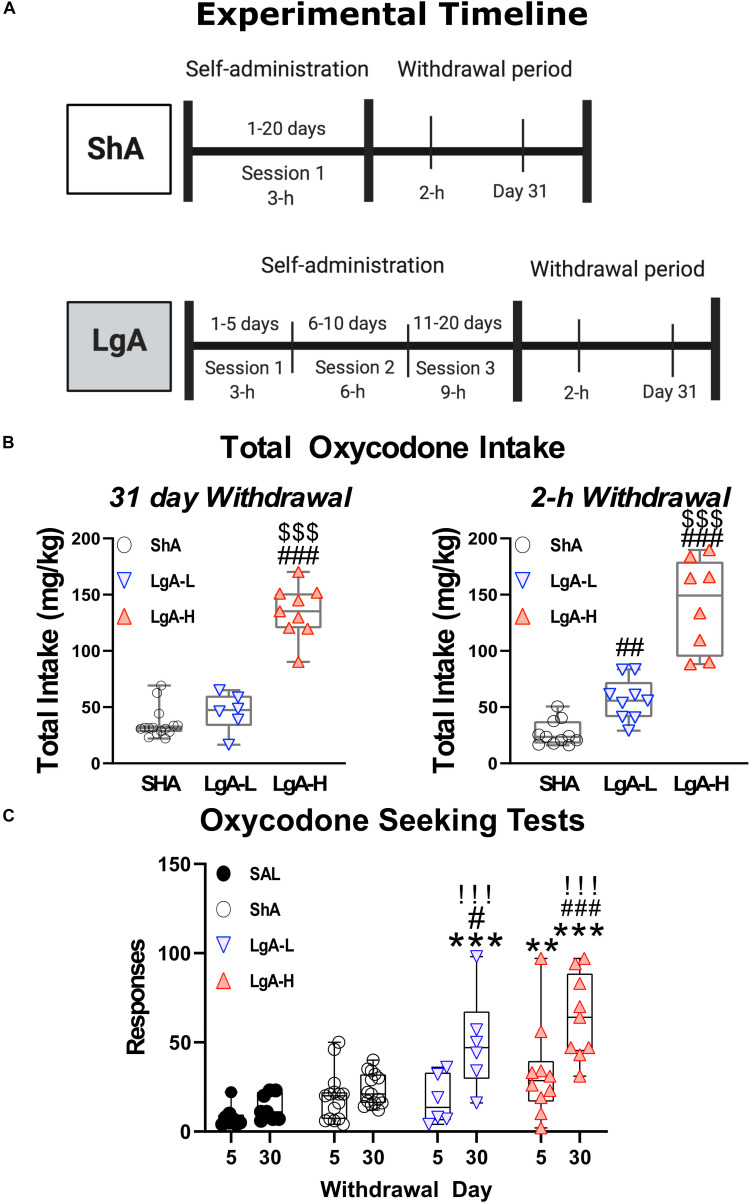
Experimental timeline and oxycodone intake by treatment group. **(A)** Experimental Timeline of oxycodone self-administration for ShA and LgA groups. Panel **(B)** shows the sum total oxycodone intake for each group from all three daily sessions over the course of the experiment. Panel **(C)** shows the lever pressing during extinction tests on day 5 and 30 of withdrawal. **, *** = *p* < 0.01, 0.001, respectively, in comparison to saline rats; #, ##, ### = *p* < 0.05, 0.01, 0.001, respectively, in comparison to ShA rats; !!! = *p* < 0.001 compared to the same treatment group on day 5.

Animals were surgically implanted with intravenous jugular catheters (Cadet et al., 2017; Blackwood et al., 2019a,b). An intraperitoneal injection of buprenorphine (0.1 mg/kg) was given to each rat to manage pain following surgery and were allowed 1 week of recovery before beginning SA. Rats were trained in SA chambers located inside sound-attenuated cabinets and controlled by a Med Associates System (Med Associates, St Albans, VT, United States). Rats were housed in these chambers for the duration of the experiment. Rats were randomly assigned to either saline (Sal) or oxycodone groups. Oxycodone-assigned rats were trained to self-administer oxycodone-HCL (NIDA Pharmacy, Baltimore, MD, United States) using short-access and LgA paradigms (Figure 1). Short-access (ShA) rats were trained for one 3-h daily session for the course of the experiment. LgA rats were trained for a single 3-h daily session during the first week of SA, two 3-h daily sessions with a 30 min break in between sessions during the second week, and three 3-h daily sessions with a 30 min break between sessions for the remainder of the SA. Lever presses were reinforced using a fixed ratio-1 with a 20-s timeout accompanied by a 5-s compound tone-light cue. Rats self-administered oxycodone at a dose of 0.1 mg/kg per infusion over 3.5-s (0.1 ml per infusion. The lever was made available and cue was presented along with an oxycodone infusion to signal the start of the session. At the end of each 3-h session and at the end of the day, the tone-light cue was turned off and the levers retracted. Saline rats were assigned to either a ShA or LgA training paradigm as well and received a 0.1 ml of 0.9% saline per infusion. After the last day of training, some rats were euthanized 2-h after the last SA session whereas other rats were returned to the animal vivarium and individually housed with no access to oxycodone during which time they participate in drug seeking tests under extinction conditions. Briefly, rats underwent 3-h cue-induced drug seeking tests on withdrawal days 5 (WD5) and 30 (WD30) during which time presentations of the cue and lever pressing were not accompanied by any oxycodone infusion.

Rats that experienced catheter failure or became sick and unable to continue in the experiment were removed from the study and excluded from further analysis.

### mRNA Extraction and Quantitative RT-PCR

Rats were euthanized either 2-h after the last SA session or 24-h after the last oxycodone seeking test. We chose the 2-h time points based on our previous experiments with methamphetamine (Cadet et al., 2014b, 2017) and oxycodone (Blackwood et al., 2020), in which we were able to identify changes in mRNAs coding for immediate early genes, potassium channels, or stress-related peptides. We chose 24 h after the drug seeking test because we assumed that most of the effects of only lever pressing would have disappeared after 24 h. Therefore, we thought it like that we would be measuring mainly the effects of prolonged oxycodone withdrawal. We have published other studies with methamphetamine using a similar approach (Daiwile et al., 2019).

The hippocampus was then dissected and isolated using coordinates (A/P −5 to −7 mm bregma, mediolateral ± to 6 mm, D/V −2 to −8 mm) according to Paxinos and Watson (1998). Collected hippocampi were then used for RNA extraction using RNeasy Mini Kit (Qiagen, Valencia, CA, United States). RNA preparation and RTqPCR experiments were performed as previously described (Cadet et al., 2017). *B2M*, a gene coding for the class I major histocompatibility complex protein β-2-microglobulin was used as reference gene, as it has been used previously in rats given opioids as well as methamphetamine (Blackwood et al., 2019a; Zoubková et al., 2019). In addition, as a matter of principle, we always make sure that the expression of any reference gene is not altered under the conditions of our experiments before using it as a reference gene. The results are shown as fold changes calculated as the ratios of normalized gene expression data for oxycodone SA groups compared to the saline group. All quantitative data are presented as means ± SEM. Primer sequences are listed in Table 1.

**TABLE 1:** Shows RT-PCR primer sequences used in our experiments.

**Gene name**	**Forward primer**	**Reverse primer**
*B2m*	GAT CTT TCT GGT GCT TGT	AGC TCA ATT TCT ATT TGA GGT
*GluA1*	GGC AAA TAC GCC TAC C	ACT CGA TTA AGG CAA CC
*GluA2*	TCC TAC ACG GCT AAC TT	GCT CGA TGT ACT CGT TC
*GluA3*	CGT GCG ATA CGA TGA A	CCA GAC CTC CGA CAA G
*GluA4*	TGA GCA ACG TAG CAG G	GTC AGG GGT AAG CAC A
*GluN1*	AAA ACA CAA TTA CGA GAG C	CCT GAT ACC GAA CCC A
*GluN2A*	AGA ATA TAA CCC TGC CTG A	GGT AAA GTG CTT GGC AT
*GluN2B*	TTC AAG CGA GAT TCG G	GGA ATT AGT CGG GCT TT
*GluN2C*	ATG TTC GTG ATG TGT CTC	TTG AAG ACC AGT GCC CA
*GluN2D*	AGT TTT CAT CTT TGA GTA CC	ATG TTG TTT TCC GTG G
*GluN3B*	ACT ATG AGG TGT CCA TAG A	ATG AAG CCT GAA GAC TTG TA
*Grm1*	GAG TTC GTG TAC GAG C	GCG TAG GTT ACA TTT GG
*Grm2*	TTC AAG ACC GCG AAG T	CGA CGA CGT TGT TGA G
*Grm3*	AAG TCC TAC GAC AGC G	CCA GGG GTT ACG ATG A
*Grm4*	CGC TAC AAC GAT ACC C	CGG TAA ATG CGG TTG G
*Grm5*	CTC AGT TAG TGA TCG CT	CTG GTC TTA AAC GCA TAG
*Grm6*	ATC GAC GGA TTT GAC C	CGC TCT ATC ACG AAC T
*Grm7*	GTT TAA TAT CGG TGC AGC	AGA TTG TAA CGC TGG T
*Grm8*	GAC TAT GGC GAA CAG C	AGT AAG TGT CGT TGT CT

*B2m* was used as a reference gene. Forward primers are complimentary to the anti-sense strand and reverse primers are complimentary to the sense strand.

### Statistical Analyses

One-Way Analysis of Variance (One-Way ANOVA) was used to analyze the PCR data with the normalized fold change in mRNA levels as the dependent variable and the treatment group (SAL, ShA, LgA-L, LgA-H) as the independent variable. Outliers were excluded according to results of the Robust regression and Outlier removal Test (ROUT) method with Q = 1%. This was followed by Tukey’s *post hoc* test or Bonferroni *post hoc* test to look for significance between groups. Regressions were performed to look for correlations between oxycodone intake and mRNA expression. The null hypothesis was rejected at *p* < 0.05. All statistical tests were performed using GraphPad Prism version 8.4.2 (GraphPad Software, San Diego, CA, United States).

## Results

### Rats Given Long-Access to Oxycodone Differentially Escalate Their Drug Intake

Figure 1 shows the experimental timeline of the SA paradigm and the total amount of oxycodone taken by each rat over the course of the experiment. Rats were either given short-access to oxycodone or LgA to oxycodone as described in the method section (Figure 1A). LgA rats take significantly more oxycodone than the ShA rats. LgA groups could be divided into two further groups, long-access high (LgA-H) and long-access low (LgA-L), based on how they escalated their intake and how much oxycodone they ended up taking (Figure 1B). Rats that took fewer than 50 infusions per day were put in the LgA-L group whereas the LgA-H consisted of rats that took more than 50 infusions per day. The LgA groups both showed incubation of craving during the drug seeking test on WD30 of withdrawal from oxycodone as reported previously (Blackwood et al., 2019a,b; Figure 1C).

### Hippocampal AMPAR Subunit mRNAs Are Differentially Regulated Following Withdrawal From Oxycodone SA

**FIGURE 2:**
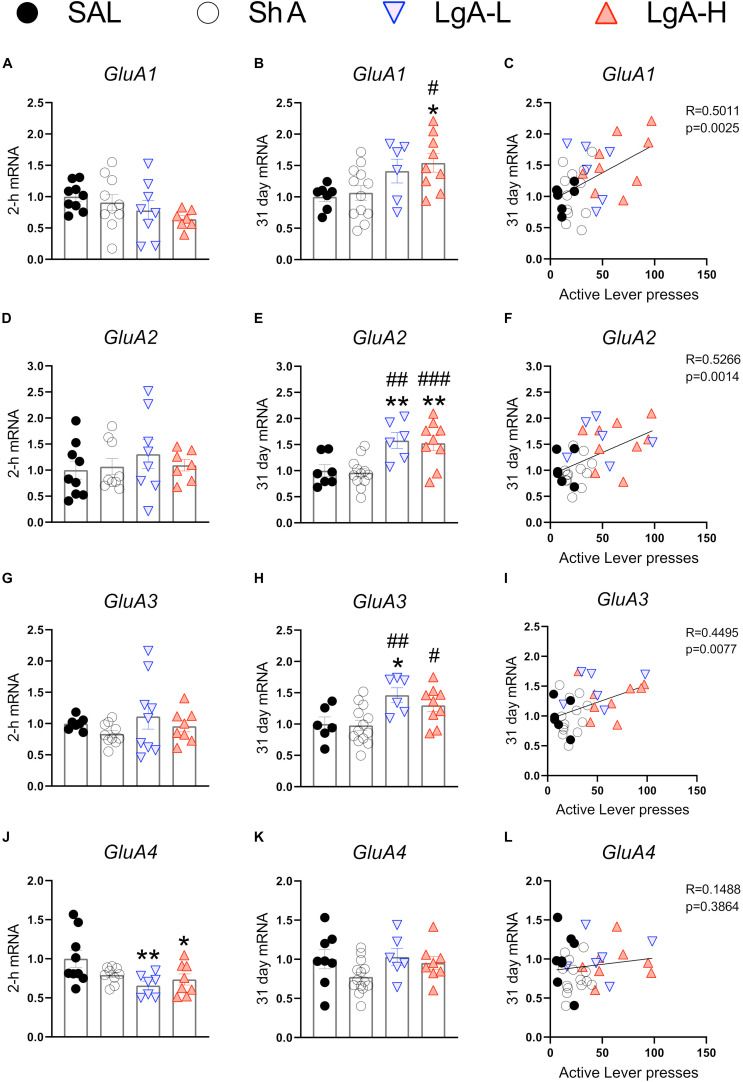
Rats undergoing long-term withdrawal from oxycodone showed increased expression of AMPA receptor mRNA. **(A–C)** *GluA1* mRNA expression is significantly increased in the LgA-H group compared to SAL at 31 days and showed a significant correlation with lever pressing on WD30. **(D–F)** *GluA2* mRNA expression is significantly increased in the LgA-L and LgA-H groups compared to the SAL and ShA groups at 31 days and showed a significant correlation with lever pressing on WD30. **(G–I)** *GluA3* mRNA expression is significantly increased in the LgA-L compared to the SAL and ShA grops as well as in the LgA-H group compared to the ShA group at 31 days and showed a significant correlation with lever pressing on WD30. **(J–L)** *GluA4* mRNA expression is significantly decreased (in the LgA-L and LgA-H group compared to SAL at 2 h and showed no significant correlation between 31 day mRNA expression and lever pressing on WD30. Key to statistics: *, **, = *p* < 0.05, 0.01, respectively, in comparison to Sal rats; #, ##, ### = *p* < 0.05, 0.01, 0.001, respectively, in comparison to ShA rats. Statistical Analyses were performed by One Way ANOVA followed by Fisher’s PLSD *post hoc* test, and correlation was tested by simple linear regression.)

α-Amino-3-hydroxy-5-methyl-4-isoxazolepropionic acid receptors participate in the regulation of neurotransmission, synaptic plasticity and LTP that are impacted by opioid exposure (Ortiz et al., 1995; Wang et al., 2004). Figure 2 shows the effects of oxycodone intake and withdrawal on *GluA1-4*. mRNA levels. There were no significant differences in *GluA1* [*F*_(3, 30)_ = 1.641, *p* = 0.2008], *GluA2* [*F*_(3, 29)_ = 0.479, *p* = 0.6994], or *GluA3* [*F*_(3, 30)_ = 1.013, *p* = 0.4006] mRNA levels between groups at 2 h after last oxycodone intake (Figures 2A,D,G). However, *GluA4* mRNA expression was significantly decreased [*F*_(3, 29)_ = 3.760, *p* = 0.0214] (Figure 2J) in the LgA groups compared to saline at that time. *GluA1* mRNA expression was significantly upregulated [*F*_(3, 30)_ = 3.730, *p* = 0.0217] (Figure 2B) in the LgA-H group compared to the ShA group and saline controls at 31 days. *GluA2* mRNA expression also showed significant increases [*F*_(3, 30)_ = 7.685, *p* = 0.0006] (Figure 2E) in both LgA groups compared to the ShA group and saline controls at 31 days. In addition, *GluA3* expression was significantly increased [*F*_(3, 30)_ = 5.000, *p* = 0.0063] (Figure 2H) in both LgA groups compared to the ShA group. In contrast, there were no significant changes in *GluA4* [*F*_(3, 30)_ = 2.108, *p* = 0.1187] expression at that time (Figure 2K). Interestingly, the changes in *GluA1*, *GluA2*, and *GluA3* mRNA levels were significantly positively correlated with increased lever pressing (incubation) after 31 days of withdrawal (Figures 2C,F,I).

### Selective Decreases in GluN Subunit Expression in LgA Rats After Drug Withdrawal

**FIGURE 3:**
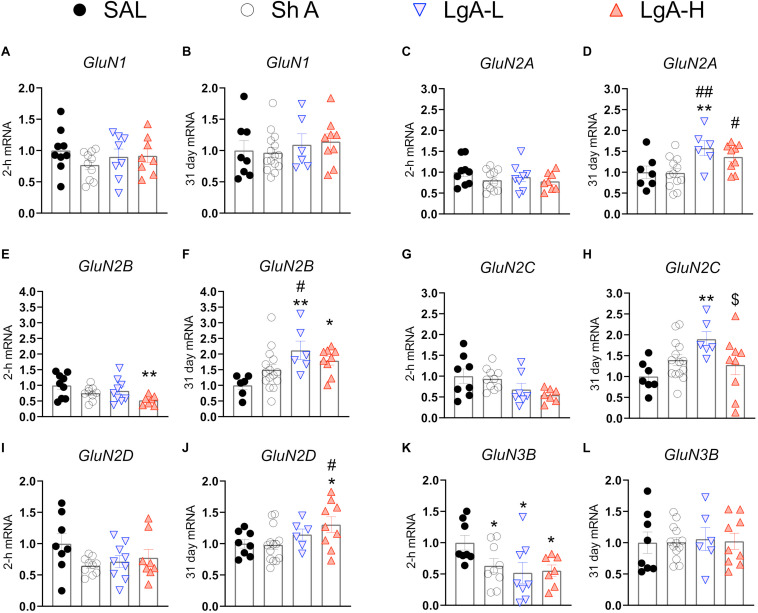
Changes in NMDA Receptor mRNA expression during oxycodone intake and withdrawal. **(A,B)** *GluN1* showed no significant changes at 2-h or 31 days. **(C,D)** *GluN2A* is significantly increased in the LgA-L group compared to the Sal and SHA groups and in the LgA-H group compared to the SHA group at 31 days. **(E)** *GluN2B* is significantly down in the LgA-H group compared to SAL at 2-h. **(F)** *GluN2B* is significantly up compared to SAL in the LgA-L and LgA-H groups and LgA-L is up compared to SHA at 31 days. **(G,H)** *GluN2C* showed no significant changes at 2-h but was significantly up in the LgA-L group compared to the SAL and LgA-H groups. **(I,J)** *GluN2D* is significantly increased in the LgA-H group compared to SAL and ShA at 31 days, but not 2-h. **(K,L)** *GluN3B* is significantly down in all groups at 2-h but not at 31 days. Key to statistics: *, **, = *p* < 0.05, 0.01, respectively, in comparison to Sal rats; #, ## = *p* < 0.05, 0.01, respectively, in comparison to ShA rats; $ = *p* < 0.05, respectively, in comparison to LgA-L rats. Statistical Analyses were performed as described in Figure 2.

N-methyl-D-aspartate receptors are also important regulators of synaptic plasticity and synaptic transmission (Lüscher and Malenka, 2012). They work in tandem with AMPA receptors to facilitate synaptic transmission and regulate LTP (Lüscher and Malenka, 2012). Figure 3 shows the effects of withdrawal from oxycodone SA on the expression of GluN subunit mRNAs. There were no significant changes in the expression of *GluN1* [*F*_(3, 31)_ = 0.9200, p = 0.4384] or *GluN2A* [*F*_(3, 32)_ = 1.111, *p* = 0.3590] mRNA at 2-h after last oxycodone intake (Figures 3A,C). *GluN2B* mRNA expression is significantly downregulated [*F*_(3, 30)_ = 3.255, *p* = 0.0353] at 2-h in the LgA-H group compared to saline only (Figure 3E). *GluN2C* mRNA levels are significantly decreased [*F*_(3, 28)_ = 2.959, *p* = 0.0494] in the LgA-H group compared to the ShA and the control groups (Figure 3G). *GluN2D* expression is unchanged [*F*_(3, 31)_ = 1.9535, *p* = 0.1445] at 2-h after last oxycodone SA session (Figure 3I). *GluN3B* mRNA expression is significantly downregulated [*F*_(3, 28)_ = 3.019, *p* = 0.0464] at 2-h in both LgA groups compared to controls (Figure 3K). *GluN1* mRNA expression was also not impacted [*F*_(3, 34)_ = 0.480, *p* = 0.6984] after 31 days of withdrawal (Figure 3B). *GluN2A* mRNA expression was significantly increased [*F*_(3, 32)_ = 4.529, *p* = 0.0093] in the LgA groups compared to the ShA group (Figure 3D). *GluN2B* mRNA expression is significantly upregulated [*F*_(3, 32)_ = 4.340, *p* = 0.0113] in LgA groups compared to the saline controls (Figure 3F). *GluN2C* mRNA is significantly increased [*F*_(3, 32)_ = 3.307, *p* = 0.0325] in the LgA-L group compared to the LgA-H group and the controls (Figure 3H). *GluN2D* mRNA is significantly increased [*F*_(3, 33)_ = 2.778, *p* = 0.0566] in the LgA-H group compared to the ShA and control groups (Figure 3J). There were no significant changes in the expression of *GluN3B* [*F*_(3, 32)_ = 0.032, *p* = 0.9923] following 31 days of withdrawal (Figure 3L). There were no significant correlations between *GluN* subunit mRNA expression and lever pressing on withdrawal day 30.

### Group I Metabotropic Glutamate Receptors Are Upregulated Following Abstinence From Oxycodone for 31 Days

**FIGURE 4:**
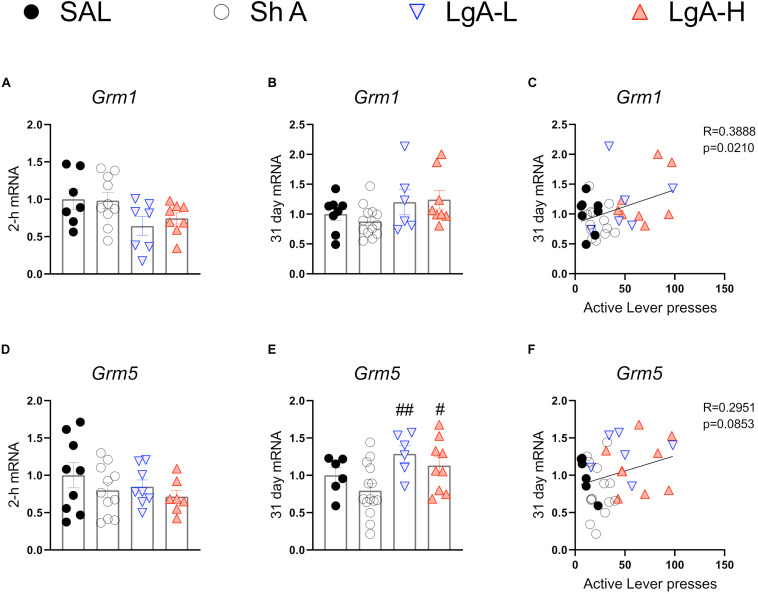
Group I metabotropic glutamate receptor mRNA expression is increased during opioid withdrawal. **(A–C)** *Grm1* showed no significant change at 2-h or 31-days after last SA, but expression at 31 days was correlated with lever pressing on WD30. **(D–F)** *Grm5* is significantly increased in both LgA groups compared to SHA group at 31 days, but mRNA expression at 31 days was not correlated with lever pressing on WD30. Key to statistics: #, ## = *p* < 0.05, 0.01, respectively, in comparison to ShA rats. Statistical Analyses were performed as described in Figure 2.

**FIGURE 5:**
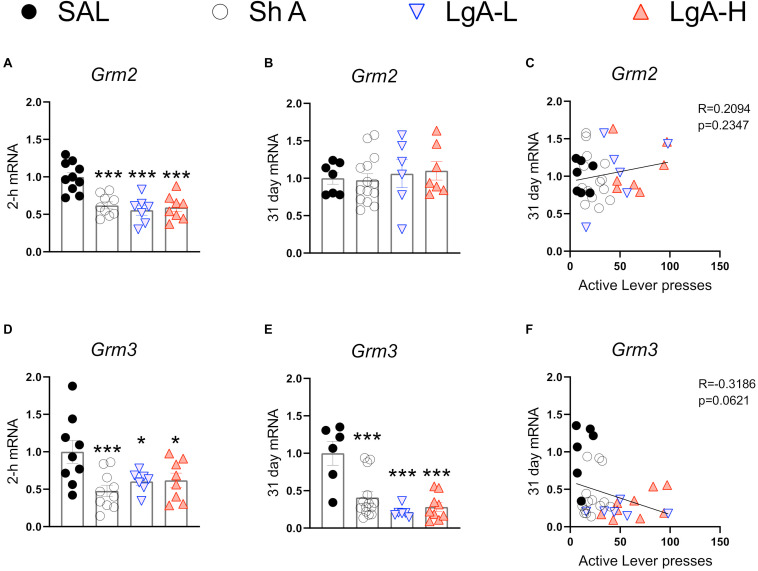
Group II metabotropic glutamate receptor mRNA expression is downregulated during oxycodone intake and maintained through long-term withdrawal. **(A–C)** *Grm2* was significantly decreased in the ShA, LgA-L, and LgA-H groups compared to SAL at 2-h but not at 31days and there was no significant correlation between lever pressing on WD30 and gene expression at 31 days. **(D–F)** Grm3 was significantly downregulated in all groups at both 2-h and 31 days but showed no significant correlation. Key to statistics: *, ***, = *p* < 0.05, 0.001, respectively, in comparison to Sal rats. Statistical Analyses were performed as described in Figure 2.

Figure 4 shows mRNA expression data for group I mGluRs. There were no significant changes in *Grm1* [*F*_(3, 28)_ = 2.526, *p* = 0.0778] or *Grm5* [*F*_(3, 31)_ = 0.959, *p* = 0.4245] expression at 2-h after the last oxycodone session (Figures 4A,C). There were also no significant changes in *Grm1* after withdrawal day 31 [*F*_(3, 31)_ = 2.062, *p* = 0.1255] (Figure 4B). There were, however, significant increases [*F*_(3, 31)_ = 3.905, *p* = 0.0178] in *Grm5* mRNA expression both LgA groups compared to the ShA group at 31 days (Figure 4E). Interestingly, changes in *Grm1* mRNA expression were positively correlated with lever pressing on WD30 (Figure 5C).

### Oxycodone SA Is Associated With Downregulated Expression of Hippocampal Group II Metabotropic Glutamate Receptors

Figure 5 shows mRNA expression data for group II mGluR mRNAs. *Grm2* [*F*_(3, 30)_ = 13.484, *p* < 0.0001] and *Grm3* [*F*_(3, 30)_ = 4.922, *p* = 0.0067] mRNA levels were significantly downregulated in all oxycodone groups compared to controls at 2-h after the last oxycodone session (Figures 5A,D). At 31 days of withdrawal, there were no significant changes in *Grm2* expression [*F*_(3, 31)_ = 0.06, *p* = 0.9618] (Figure 5B). In contrast, *Grm3* mRNA expression remains significantly downregulated [*F*_(3, 31)_ = 11.991, *p* < 0.0001] in all oxycodone groups (Figure 5E). Interestingly, changes in *Grm3* expression showed significant negative correlation with lever pressing on withdrawal day 31 (Figure 5F).

### Group III Metabotropic Glutamate Receptors Are Upregulated in the Hippocampus Following Oxycodone Withdrawal

**FIGURE 6:**
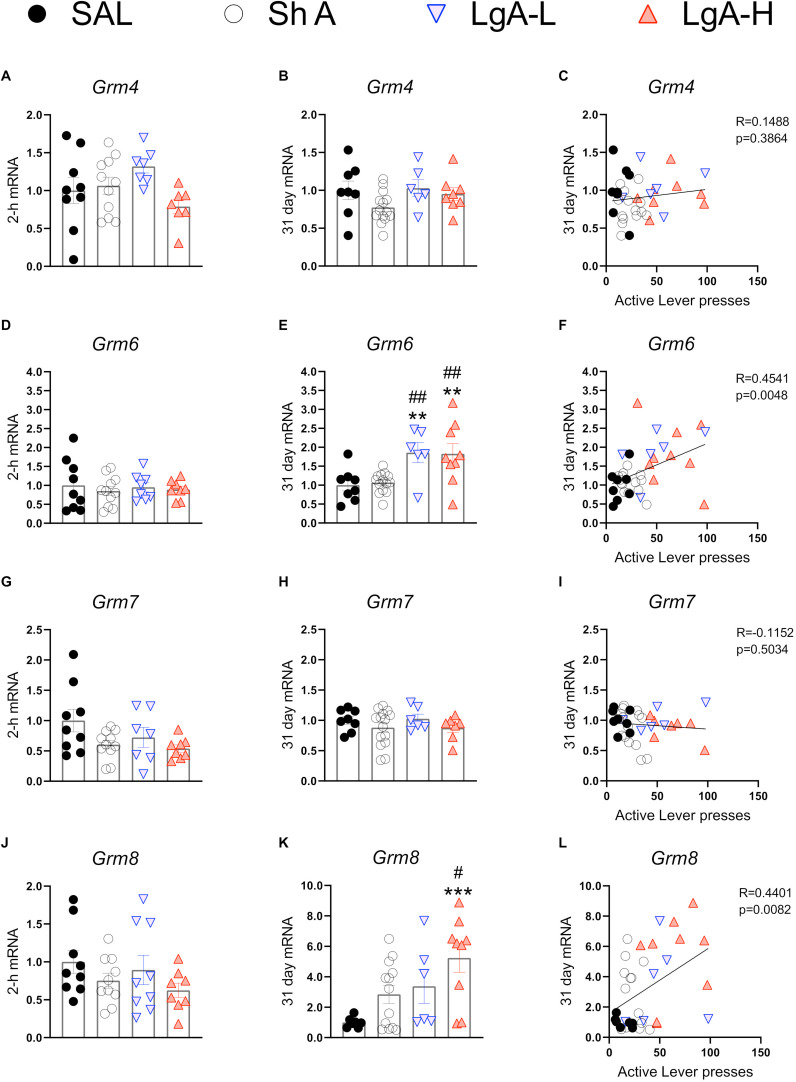
Group III metabotropic glutamate receptor mRNA expression is upregulated during withdrawal from oxycodone. **(A–C)** There were no significant changes in mRNA expression at 2-h or 31 days and no significant correlation between 31-day mRNA expression and lever pressing on WD30. **(D–F)** *Grm6* is significantly upregulated in both LgA groups compared to ShA and SAL at 31 days and there is a significant positive correlation between lever pressing on WD30 and *Grm6* expression at 31 days. **(G–I)** There were no significant changes in *Grm7*. **(J–L)** *Grm8* is significantly upregulated in the LgA-H group compared to ShA and SAL at 31 days and is positively correlated with lever pressing on WD30. Key to statistics: **, ***, = *p* < 0.01, 0.001, respectively, in comparison to Sal rats; #, ## = *p* < 0.05, 0.01, respectively, in comparison to ShA rats. Statistical Analyses were performed as described in Figure 2.

Figure 6 show mRNA expression data for group III mGluRs (Alexander et al., 2019). These receptors including mGluR6 receptors occur in the brain (Huang et al., 2012; Palazzo et al., 2020) and serve to suppress glutamate release (Schoepp, 2001; Niswender and Conn, 2010). There were no changes in *Grm4* [*F*(3, 30) = 2.372, *p* = 0.0901], *Grm6* [*F*_(3, 32)_ = 0.2012, *p* = 8948], *Grm7* [*F*_(3, 31)_ = 2.631, *p* = 0.0675], or *Grm8* [*F*_(3, 32)_ = 1.275, *p* = 0.2997] (Figures 6A,D,G,J) at 2-h after the last oxycodone SA session.

There were also no significant changes in *Grm*4 [*F*_(3,32)_ = 2.108, *p* = 0.1187] during late withdrawal (Figure 6B). However, there were significant increases in *Grm6* mRNA expression [*F*_(3, 33)_ = 6.634, *p* = 0.0012] in both LgA groups compared to the other groups (Figure 6E). *Grm7* mRNA expression shows no significant changes [*F*_(3,33)_ = 0.882, *p* = 0.4608] at that time (Figure 6H) whereas *Grm8* mRNA expression was significantly increased [*F*_(3, 31)_ = 4.822, *p* = 0.0072] in the LgA-H group compared to controls (Figure 6K). Changes in *Grm6* (Figure 6F) and mGluR8 (Figure 6L) mRNA levels were both positively correlated with lever pressing measured on WD30.

## Discussion

Oxycodone misuse and its many medical complications have made significant contributions to the current opioid public health crisis in the USA (Boscarino et al., 2010; Rudd et al., 2016). Although efforts have been made to develop more effective treatments against opioid addiction, much more remains to be done in order to understand the biochemical and molecular effects of chronic exposure to and withdrawal from opioid drugs on the brain. Toward that end, we have been investigating the biochemical and molecular consequences of exposure to oxycodone in a rat model of drug SA (Blackwood et al., 2019a). We found that rats given LgA to oxycodone escalate in their drug taking and show incubation of drug seeking following a 31-day withdrawal period (Blackwood et al., 2019a). We have also shown that oxycodone SA-related behaviors are associated with significant changes in the expression of opioid receptor genes in the dorsal striatum and hippocampus of these rats (Blackwood et al., 2019a). The observation of increased oxycodone drug seeking after 31 days of withdrawal had suggested the possibility that genes involved in hippocampal memory processes might also be impacted in these animals. We thus tested the possibility that the mRNA expression of AMPAR subunits, NMDAR subunits, and mGluRs might be altered in the hippocampi of rats that were exposed to oxycodone at two time points following withdrawal. We found, importantly, that most of the changes in gene expression occurred after WD30 as reported in the results.

The observations of increased expression of AMPAR subunits, *GluA1*, *GluA2*, and *GluA3* mRNA levels in the LgA-H groups that showed incubation of oxycodone craving suggest the potential involvement of these glutamate receptors in the incubation phenomenon. This suggestion is supported, in part, by the fact that changes in *GluA1*, *GluA2*, and *GluA3* mRNA levels were positively correlated with increased lever pressing (incubation) after WD30. Given the well-established role of AMPA receptors in synaptic plasticity (Diering and Huganir, 2018), our results suggest a mechanism via which increased expression of AMPA receptors might enhance cue-induced oxycodone seeking because of strengthened synaptic connections in the hippocampus during long-term withdrawal from oxycodone. The proposition of the involvement of these receptors in oxycodone craving is also consistent with previous studies that had reported increased expression of AMPA receptors in the nucleus accumbens of rats had exhibited incubation of cocaine (Conrad et al., 2008; Mameli et al., 2009; McCutcheon et al., 2011) or methamphetamine (Scheyer et al., 2016; Murray et al., 2019) craving. It is important to note that although the relative changes in expression of *GluA1*, *GluA2*, and *GluA3* were approximately the same in the LgA groups, there still might be distinct changes in receptor compositions that occur after translation and/or during assembly of these receptors that cannot be assessed by measuring only gene expression. These changes may include increased differential expression of homomeric GluA1 AMPARs (GluA2-lacking) that are calcium permeable (Hollmann et al., 1991). These compositions are known to be accompanied by enhanced AMPAR neurotransmission (Churchill et al., 1999; Mameli et al., 2009). Importantly, Ping et al. (2008) reported that injections of antisense oligonucleotides directed against GluA1 into the NAc attenuated cocaine-primed reinstatement. Therefore, it is not far-fetched to suggest that differential compositions of AMPA receptors might modify hippocampal programs that might enhance oxycodone seeking during prolonged withdrawal from oxycodone SA. This discussion supports the need to examine changes in protein compositions of hippocampal AMPA receptors in follow-up studies of oxycodone SA and withdrawal. When taken together with the studies of the incubation phenomenon in rats that self-administered cocaine (Conrad et al., 2008) or methamphetamine (Scheyer et al., 2016), the present study suggests a potential role of anti-AMPAR receptor drugs in the treatment of SUDs to prevent relapses.

In addition to the changes in expression of *GluA* subunits in the hippocampus, we also measured potential changes in other glutamate receptors in the hippocampus after oxycodone withdrawal. It has indeed been suggested that NMDA receptor subunits might play some roles in various aspects of addiction (Hopf, 2017; Smaga et al., 2019). We found few changes in the expression of *GluN* mRNAs in rats euthanized at the 2-hr time point. However, when compared to control and ShA rats, there were significant increases in *GluN2A* and *GluN2B* mRNA levels in both LgA groups that showed incubation of oxycodone seeking. These observations are consistent with suggestions that NMDA receptors participate in the behavioral effects of alcohol (Morisot and Ron, 2017). Specifically, Follesa and Ticku (1995) had reported increased *GluN2A* and *GluN2B* in the hippocampi of rats chronically administered ethanol. Kalluri et al. (1998) also reported increased GluN2A and GluN2B protein levels after chronic alcohol. However, because these changes were measured only for 48 h after cessation of alcohol intake, it is not clear what would have happened after 30 days of withdrawal. Our results are also consistent with those of Ma et al. (2007) who reported that intra-hippocampal injection of a GluN2B inhibitor, ifenprodil, was able to attenuate morphine-induced reinstatement of extinguished morphine conditioned place preference. Escalating doses of cocaine also caused increased GluN*2B* mRNA and protein levels in the hippocampus of mice engaged in a CPP paradigm (Liddie and Itzhak, 2016). Withdrawal from cocaine SA is accompanied by increased GluN2A protein expression but a potential relationship between these changes and cocaine seeking was not discussed (Pomierny-Chamiolo et al., 2015). Studies investigating the role of other GluN subunits are very scarce and the relationship of changes in NMDA receptor compositions to cue- or context-induced drug seeking remain to be fully investigated, a line of queries that might prove to be potentially fruitful.

Our study also documented some changes in the expression of metabotropic receptors during withdrawal from oxycodone SA. Of the type I mGluRs, *Grm5* mRNA levels were increased at WD30 without there being any relationship to incubation of oxycodone seeking. Although chronic intrathecal injections of morphine also caused increased mGluR5 protein expression in the frontal cortex of mice euthanized after the last of 5 injections (Huang et al., 2019), studies on the role of this subunit on cue-induced drug seeking is non-existent. We also documented decreased mRNA levels in *Grm2* and *Grm3* mRNA levels in all rats exposed to oxycodone, suggesting profound inhibitory effects of oxycodone on those subunits. The effects of oxycodone on *Grm2* mRNA were long-lasting since they were still present even after WD30. The fact that *Grm3* mRNA levels returned to normal levels suggests that the two genes are regulated differentially by oxycodone. Our findings are consistent with the report of decreased mGluR2/3 protein expression in the nucleus accumbens following withdrawal from repeated subcutaneous injection of morphine (Qian et al., 2019). Because mGluR2/3 receptors are located predominantly on pre-synaptic axonal domains and glutamate terminals in the hippocampus (Petralia et al., 1996; Ohishi et al., 1998) and serve to suppress glutamate release (Schoepp, 2001; Niswender and Conn, 2010), it is possible that oxycodone-induced decrease in the expression might be compensatory in response to oxycodone-associated increased glutamate release during the drug SA experiment. Interestingly, activation of mGluR2/3 receptors by their agonist, LY379268, has been reported to attenuate reinstatement of cue-induced heroin seeking (Bossert et al., 2005). Moreover, activation of group II metabotropic hippocampal glutamate receptors can attenuate cue-induced seeking in rats trained to self-administer ethanol (Zhao et al., 2006). The positive modulation of mGluR2/3, LY37968, also reduced cue-induced methamphetamine seeking after prolonged withdrawal (Kufahl et al., 2013). Together, these studies implicate mGluR2/3 in the molecular mechanisms involved in promoting relapse after abstinence from drug taking.

We found that *Grm8* mRNA levels were increased in the LgA-H rats whereas *Grm6* was increased in all the LgA rats. Similar to group II metabotropic receptors, group III mGluRs, including mGluR6 (Huang et al., 2012; Palazzo et al., 2020), are located mainly in presynaptic active zones in the brain (Ferraguti and Shigemoto, 2006; Mercier and Lodge, 2014). The recent review (Palazzo et al., 2020) of mGluR6 expression provides details about its presence beyond the visual system (Vardi et al., 2000). The increased expression of *Grm6* mRNA levels in the LgA rats and the relationship of these increases to oxycodone seeking cement an important role for these receptors in relapse to oxycodone abuse. The increases in *Grm8* mRNA expression also correlated with incubation of oxycodone craving, thus implicating both members of metabotropic glutamate type III receptors in that behavioral phenomenon. Specific genetic manipulations of mGluR6 and mGluR8 should help to establish the extent to which these genes are involved in either cue- or context-induced drug seeking. Although there are, at present, very few studies have investigated potential roles of these type III metabotropic receptors in animal models of addiction, our data are consistent with those of Nielsen et al. (2008) who were able to provide evidence that metabotropic receptors, mGluR6 and mGluR8, were correlated with the risk of developing heroin addiction in a genome-wide association study of 110 heroin addicted individuals.

In summary, we found that there were significant changes in the expression of mRNA for several glutamate receptors in the hippocampus and that some of these changes correlated positively with increased oxycodone seeking within their same individual cages at WD30, a phenomenon that may reflect relapse potential in humans under similar conditions. However, as changes in mRNA do not necessarily reflect changes in protein (Maier et al., 2009), follow up studies are needed to confirm that mRNA results translate to changes in protein expression. Because the hippocampus plays an important role in the induction of context-associated drug seeking in animal models of psychostimulants and opioids (Taubenfeld et al., 2010; Bossert et al., 2016; Galinato et al., 2018; Noe et al., 2019; Felipe et al., 2021), it will be important to investigate context- and cue-induced in parallel to assess if similar or distinct molecular changes are associated with these behavioral phenomena. In addition, although we have discussed the molecular changes in terms of their facilitating oxycodone drug seeking behaviors, it is possible that these changes might have actually been consequences to lever pressing. We think that this is unlikely because we euthanized the rats 24 h after the last drug seeking test. Moreover, our results are consistent with those of other investigators who have implicated some of these glutamate receptors in mediating drug seeking behaviors (Wolf, 2016). Nevertheless, it will be important to investigate the effects of prolonged drug withdrawal in the absence of drug seeking tests. In any case, our results are consistent with the proposal that glutamatergic and memory systems might play important roles in the manifestations and clinical course of opioid use disorders (Heinsbroek et al., 2020). The present observations broadened our insight into potential ways that glutamate receptors might act to promote incubation of oxycodone seeking after prolonged withdrawal. Dissecting these mechanisms better should help the development of novel targets for oxycodone addiction. When taken together with previous results with cocaine and methamphetamine withdrawal, our observations hint to the use of AMPAR antagonist and mGluR agonist in a general approach to therapeutic interventions against SUDs.

## Data Availability Statement

The raw data supporting the conclusions of this article will be made available by the authors, without undue reservation.

## Ethics Statement

The animal study was reviewed and approved by National Institute on Drug Abuse Institutional Animal Care and Use Committee (ACUC).

## Author Contributions

CB performed self-administration experiments. AS performed RT-PCR experiments. JC supervised the overall project. All authors prepared the manuscript.

## Conflict of Interest

The authors declare that the research was conducted in the absence of any commercial or financial relationships that could be construed as a potential conflict of interest.
